# PDGF-BB regulates splitting angiogenesis in skeletal muscle by limiting VEGF-induced endothelial proliferation

**DOI:** 10.1007/s10456-018-9634-5

**Published:** 2018-07-16

**Authors:** R. Gianni-Barrera, A. Butschkau, A. Uccelli, A. Certelli, P. Valente, M. Bartolomeo, E. Groppa, M. G. Burger, R. Hlushchuk, M. Heberer, D. J. Schaefer, L. Gürke, V. Djonov, B. Vollmar, A. Banfi

**Affiliations:** 1 Department of Biomedicine, Basel University Hospital, University of Basel, Hebelstrasse 20, 4031 Basel, Switzerland; 2 Department of Surgery, University Hospital, Basel, Switzerland; 3 Institute for Experimental Surgery, University of Rostock, Rostock, Germany; 4 Present Address: The Biomedical Research Centre, The University of British Columbia, Vancouver, Canada; 5 Institute of Anatomy, University of Bern, Bern, Switzerland

**Keywords:** VEGF, PDGF-BB, Intussusception, Vascular splitting, Shear stress, Pericytes

## Abstract

VEGF induces normal or aberrant angiogenesis depending on its dose in the microenvironment around each producing cell in vivo. This transition depends on the balance between VEGF-induced endothelial stimulation and PDGF-BB-mediated pericyte recruitment, and co-expression of PDGF-BB normalizes aberrant angiogenesis despite high VEGF doses. We recently found that VEGF over-expression induces angiogenesis in skeletal muscle through an initial circumferential vascular enlargement followed by longitudinal splitting, rather than sprouting. Here we investigated the cellular mechanism by which PDGF-BB co-expression normalizes VEGF-induced aberrant angiogenesis. Monoclonal populations of transduced myoblasts, expressing similarly high levels of VEGF alone or with PDGF-BB, were implanted in mouse skeletal muscles. PDGF-BB co-expression did not promote sprouting and angiogenesis that occurred through vascular enlargement and splitting. However, enlargements were significantly smaller in diameter, due to a significant reduction in endothelial proliferation, and retained pericytes, which were otherwise lost with high VEGF alone. A time-course of histological analyses and repetitive intravital imaging showed that PDGF-BB co-expression anticipated the initiation of vascular enlargement and markedly accelerated the splitting process. Interestingly, quantification during in vivo imaging suggested that a global reduction in shear stress favored the initiation of transluminal pillar formation during VEGF-induced splitting angiogenesis. Quantification of target gene expression showed that VEGF-R2 signaling output was significantly reduced by PDGF-BB co-expression compared to VEGF alone. In conclusion, PDGF-BB co-expression prevents VEGF-induced aberrant angiogenesis by modulating VEGF-R2 signaling and endothelial proliferation, thereby limiting the degree of circumferential enlargement and enabling efficient completion of vascular splitting into normal capillary networks despite high VEGF doses.

## Introduction

Coronary and peripheral artery diseases (CAD and PAD) remain the major causes of morbidity and mortality in Western countries, despite current medical and surgical options [1]. Therapeutic angiogenesis aims at restoring the blood supply in ischemic tissues by delivering factors that control vascular growth and is an attractive strategy to improve treatment of these disabling and often fatal conditions. Vascular Endothelial Factor-A (VEGF) is the master regulator of vascular growth both in development and tissue repair [2] and is therefore the key molecular target for therapeutic angiogenesis [3]. However, clinical trials of VEGF gene delivery have yielded disappointing results, despite the clear biological activity of the factor [3, 4]. There is therefore a clear need to better understand the mechanisms of VEGF-induced angiogenesis, particularly under therapeutically relevant conditions of factor delivery.

The best-studied mechanism of angiogenesis is sprouting, whereby specialized endothelial tip cells sense a VEGF gradient and migrate towards its source to invade surrounding tissue, followed by proliferating stalk cells that form the new vessel lumen [2]. On the other hand, expansion of already formed vascular networks can also take place by the alternative mechanism of splitting angiogenesis, which entails the formation of intraluminal endothelial pillars that split pre-existing vessels longitudinally to form new ones [5]. However, the regulation of splitting angiogenesis is little studied and still poorly understood. In order to investigate how VEGF gene delivery regulates vascular growth in the therapeutic target tissue of skeletal muscle, we have previously developed a highly controlled gene delivery platform, based on monoclonal populations of transduced myoblasts that ensure homogeneous expression of specific doses or combinations of angiogenic factors [6, 7] and we found that (1) VEGF induces either physiological microvascular networks or aberrant angioma-like structures depending on its dose localized in the microenvironment around each producing cell in vivo [7]; (2) at the doses required for functional ischemia relief [8], VEGF induces muscle angiogenesis essentially without sprouting and rather by vascular splitting [9]; and (3) the switch from normal to aberrant angiogenesis does not depend exclusively on VEGF dose, but rather on the balance between endothelial stimulation by VEGF and pericyte recruitment by Platelet-Derived Growth Factor-BB (PDGF-BB), such that PDGF-BB co-delivery ensures normal and functional microvascular growth despite high or uncontrolled VEGF expression [6, 10]. VEGF-induced angiogenesis in skeletal muscle entailed two distinct phases, namely, an initial stage of circumferential vascular enlargement during the first 4 days, followed by longitudinal splitting that is complete within day 7 [9].

We have previously established that endogenous PDGF-BB is required to ensure physiological angiogenesis by moderate VEGF doses and its co-delivery can normalize angiogenesis by high levels of VEGF, expanding its therapeutic window [6, 10]. However, how this happens and whether PDGF-BB may regulate splitting angiogenesis has not been studied. Here, we sought to investigate the cellular mechanisms by which PDGF-BB regulates the VEGF dose-dependent switch from normal to aberrant angiogenesis in skeletal muscle. We found that PDGF-BB prevents pericyte loss induced by high VEGF and regulates splitting angiogenesis by reducing the activation of VEGF-R2 signaling and endothelial proliferation, thereby limiting the degree of initial vascular enlargement and enabling successful splitting regardless of VEGF dose.

## Results

### PDGF-BB accelerates splitting angiogenesis by high VEGF

**Fig. 1:**
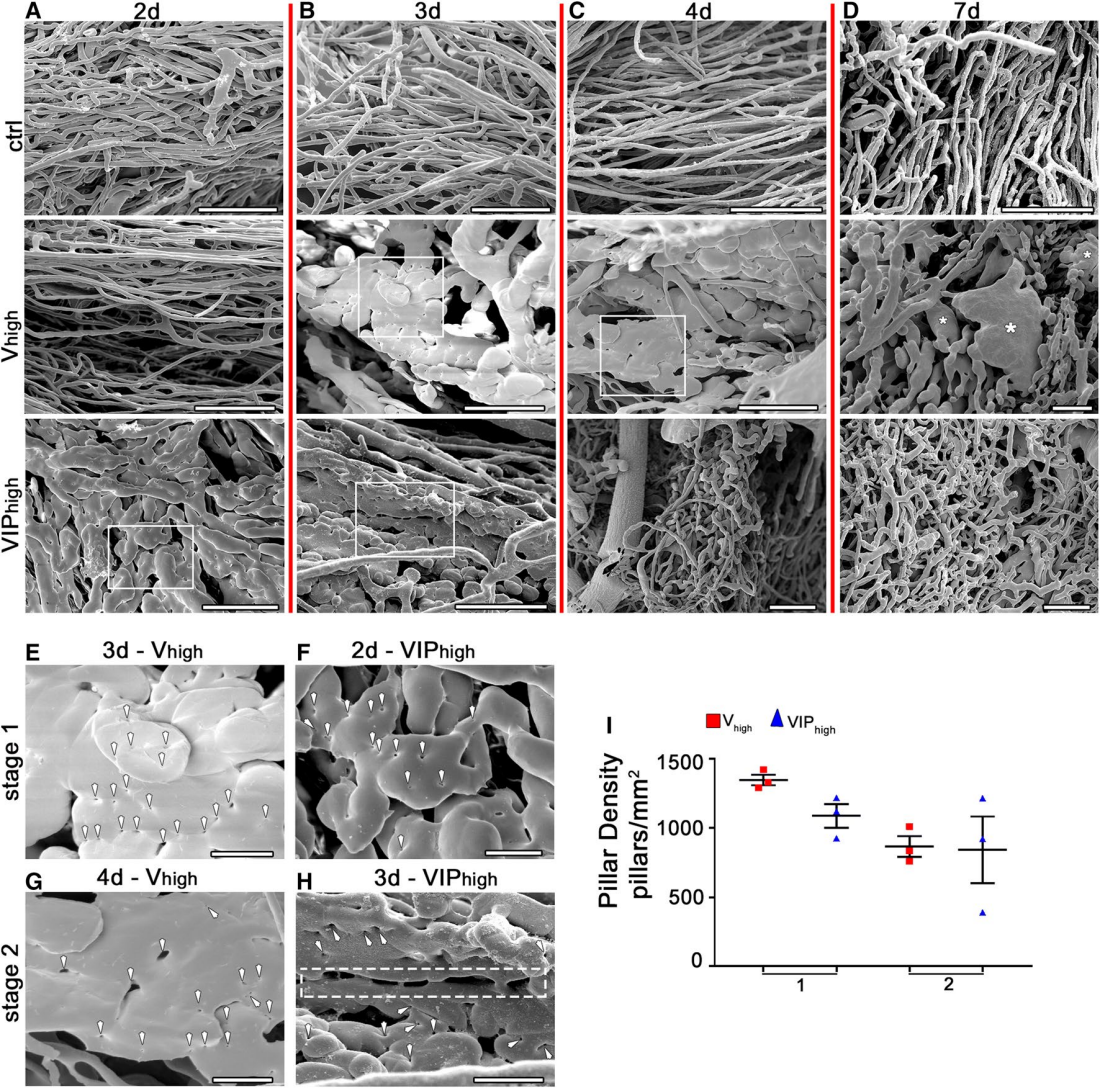
PDGF-BB co-expression anticipates vascular enlargement formation and accelerates vascular splitting: clonal populations of transduced myoblasts expressing high levels of VEGF alone (*V*_high_ myoblast) or co-expressed together with PGF-BB (VIP_high_ myoblast) and control myoblasts (ctrl) were implanted in TA and GC muscles of SCID mice. **a–h** Vascular corrosion casts of the entire legs were performed at 2, 3, 4, and 7 days post-implantation. The time point labels (2, 3, 4, and 7 days) refer to all images in each column delimited by the red vertical lines. The white rectangles in panels **b**-*V*_high_, **c**-*V*_high_, **a**-VIP_high_, and **b**-VIP_high_ are shown at higher magnification in **e–h**. White arrowheads: small indentations and holes indicative of transluminal tissue pillar formation. Dash rectangle in **h** vascular splitting after transluminal pillar fusion. Asterisks in **d**-*V*_high_ angioma-like structures devoid of further signs of pillar formation. *n* = 3 independent samples per group, per time point. **a**–**d** Scale bars = 100 µm, **e**–**h** Scale bars = 25 µm. **i** Quantification of the relative numerical pillar density (number of pillars per vessel area) at equivalent biological stages; data points represent the means of individual measurements in each sample, while bars show the overall mean ± SEM. *n* = 3 independent samples per group, per time point

First we sought to determine the cellular mechanism by which PDGF-BB co-expression prevents VEGF-induced aberrant angiogenesis, i.e., whether by switching vascular growth from splitting to sprouting, or rather modulating the initial morphogenic events of VEGF-induced vascular enlargement and splitting. In order to rigorously control the in vivo dose of VEGF and PDGF-BB, we took advantage of well characterized libraries of monoclonal populations of retrovirally transduced murine myoblasts that we previously generated and that homogeneously express specific levels of VEGF_164_ alone (V clones) or together with PDGF-BB at a fixed ratio of 1:3 (VIP clones), or PDGF-BB alone (P clones) [6, 7, 11]. As every cell in each monoclonal population has the same copy number and genomic integration sites of the retroviral vector, they all secrete the same amount of the factors and therefore the use of such populations enables the control of their dose in the microenvironment around each cell [6, 7]. Myoblast clones were selected that secrete a high level of VEGF alone (*V*_high_, causing aberrant angiogenesis) or together with PDGF-BB (VIP_high_, causing instead its normalization into physiological capillary networks) [6] and implanted them into limb muscles of mice (tibialis anterior and gastrocnemius). Myoblasts that do not express either VEGF or PDGF-BB were used as control (ctrl). The kinetics of initial vascular morphogenesis was analyzed 2, 3, 4, and 7 days after cell implantation by corrosion casting, the gold standard to identify intussusception [12], and immunofluorescence confocal microscopy, which effectively detects endothelial sprouting. Control myoblasts did not induce angiogenesis at any time point (Fig. 1a–d, ctrl). As previously observed [9], after 2 days high levels of VEGF alone did not yet perturb the pre-existing vessels (Fig. 1a, *V*_high_) and enlarged vascular structures appeared by 3 and 4 days (Fig. 1b, c, *V*_high_). Vessel casts of these structures were pierced by numerous small holes (Fig. 1b, c, *V*_high_, and arrowheads in Fig. 1e, g), which are the hallmark of the initial formation of transluminal pillars [5, 9]. Again consistently with previous observations, by 7 days signs of pillar formation had disappeared from the aberrant bulbous angioma-like structures induced by high VEGF (stars in Fig. 1d, *V*_high_). On the other hand, co-delivery of PDGF-BB caused vascular enlargements already by 2 days, whose casts also showed signs of numerous intraluminal pillars being formed (Fig. 1a, VIP_high_ and f). By day 3, vascular casts displayed both pillar formation and evidence of longitudinal splitting (Fig. 1b, VIP_high_ and dashed rectangle in Fig. 1h) into new vascular segments. Surprisingly, with co-delivery of high levels of VEGF and PDGF-BB vascular remodeling was already complete by 4 days, yielding networks of normal capillaries (Fig. 1c, VIP_high_), which were similar to and denser than networks visible in control samples (Fig. 1a–d, ctrl), with no further change in morphology by day 7 (Fig. 1d, VIP_high_). The occurrence of intussusceptive angiogenesis was quantified by measuring the numerical density of pillars, defined as the total number of pillars per mm^2^ of vascular surface area. Since VIP conditions displayed an anticipated and faster kinetics of vascular remodeling, we sought to compare equivalent biological stages of vessel development rather than the same time points. Therefore, we defined two different stages to represent equivalent biological states (Fig. 1e–h), i.e., stage 1 to be the first day of vascular enlargement (corresponding to day 3 for *V*_high_ and day 2 for VIP_high_ conditions) and stage 2 to be the second day of remodeling (corresponding to day 4 for *V*_high_ and day 3 for VIP_high_). As shown in Fig. 1i, quantifications confirmed the abundant and similar pillar formation during the two different stages in both conditions (day 3 *V*_high_ = 1345 ± 195 vs. day 2 VIP_high_ = 1091 ± 131, *p* = n.s.; day 4 *V*_high_ = 868 ± 74 vs. day 3 VIP_high_ = 843 ± 241, *p* = n.s.), suggesting that PDGF-BB co-delivery did not influence the timing and frequency of pillar formation.

**Fig. 2:**
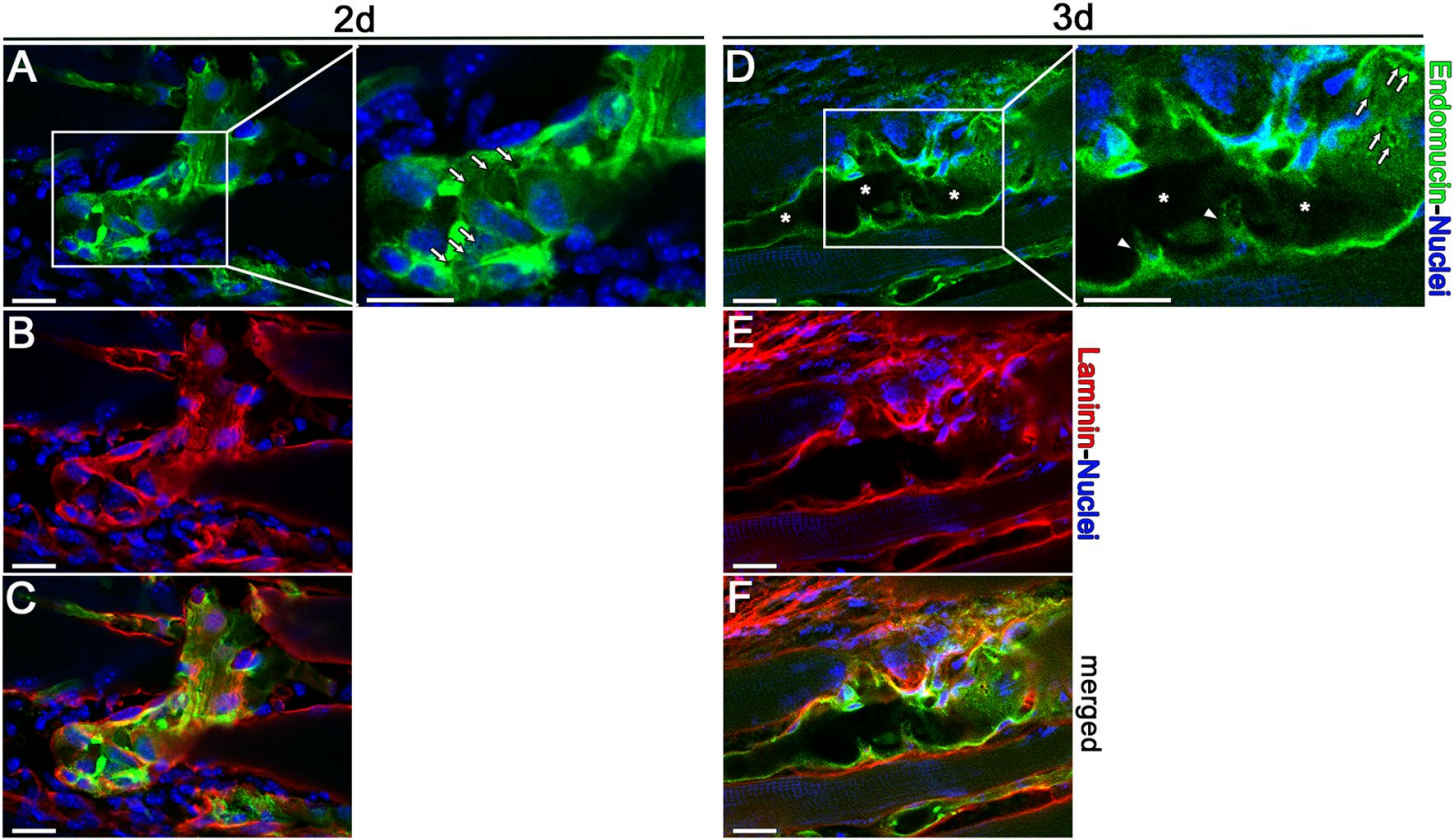
Absence of abluminal endothelial sprouting. Immunostaining with antibodies against endomucin (endothelial cells, green), laminin (basal lamina, red), and with DAPI (nuclei, blue) was performed on cryosections of TA and GC muscles 2 (**a**–**c**) and 3 days (**d**–**f**) after implantation with VIP_high_ myoblast clone. Enlarged vessels displayed no evidence of abluminal endothelial cell processes sprouting outside the basal lamina, but rather appeared pierced by numerous transluminal holes (white arrows in high-magnification panels in **a** and **d**) and displayed intraluminal vascular ridges (white arrowheads in high-magnification panel in **d**). *Vascular lumen. *n* = 2 muscles per time point; scale bars = 20 µm in all panels

On the other hand, corrosion casting only shows the lumen of vascular structures and is not optimal to detect endothelial sprouts, which are devoid of lumen in their migrating tips [13]. Therefore, we investigated the evidence for sprout formation also by confocal microscopy and 3D reconstruction of thin optical sections after immunostaining for endomucin, which homogeneously stains all endothelial structures, including the filopodia on sprouting tip cells, and laminin, which labels the basal lamina and defines the external boundary of vessels [9]. Careful analysis of the enlarged vessel walls revealed that the endomucin-positive cells of enlarged vascular structures were completely contained within the respective basal lamina and no endothelial extensions could be seen protruding outside of it, thereby confirming the lack of abluminal sprouting (Fig. 2a–c, d–f). Remarkably, in the enlarged vascular structures it was possible to observe pillar formation both as small holes in the endomucin-positive endothelium (arrows in Fig. 2a, d, high-magnification panels), compatible with those displayed in the corrosion casts, as well as endothelial filopodial extensions (arrowheads in Fig. 2d, high-magnification panel) projecting inside the vascular lumen (marked with stars). The abluminal side of both the holes (inner part) and of the intraluminal filopodia-bearing endothelial cells (external part) showed positive laminin signal, as expected for a forming intraluminal pillar [14].

Taken together, these data show that, compared to high levels of VEGF alone, PDGF-BB co-delivery: (a) did not promote endothelial sprouting, but rather modulated VEGF-induced splitting angiogenesis; (b) anticipated the initiation of vascular enlargement; (c) did not affect the timing and frequency of transluminal pillar formation; and (d) accelerated the splitting of enlarged vessels and the completion of their remodeling.

### PDGF-BB limits the degree of vascular enlargement by high VEGF

**Fig. 3:**
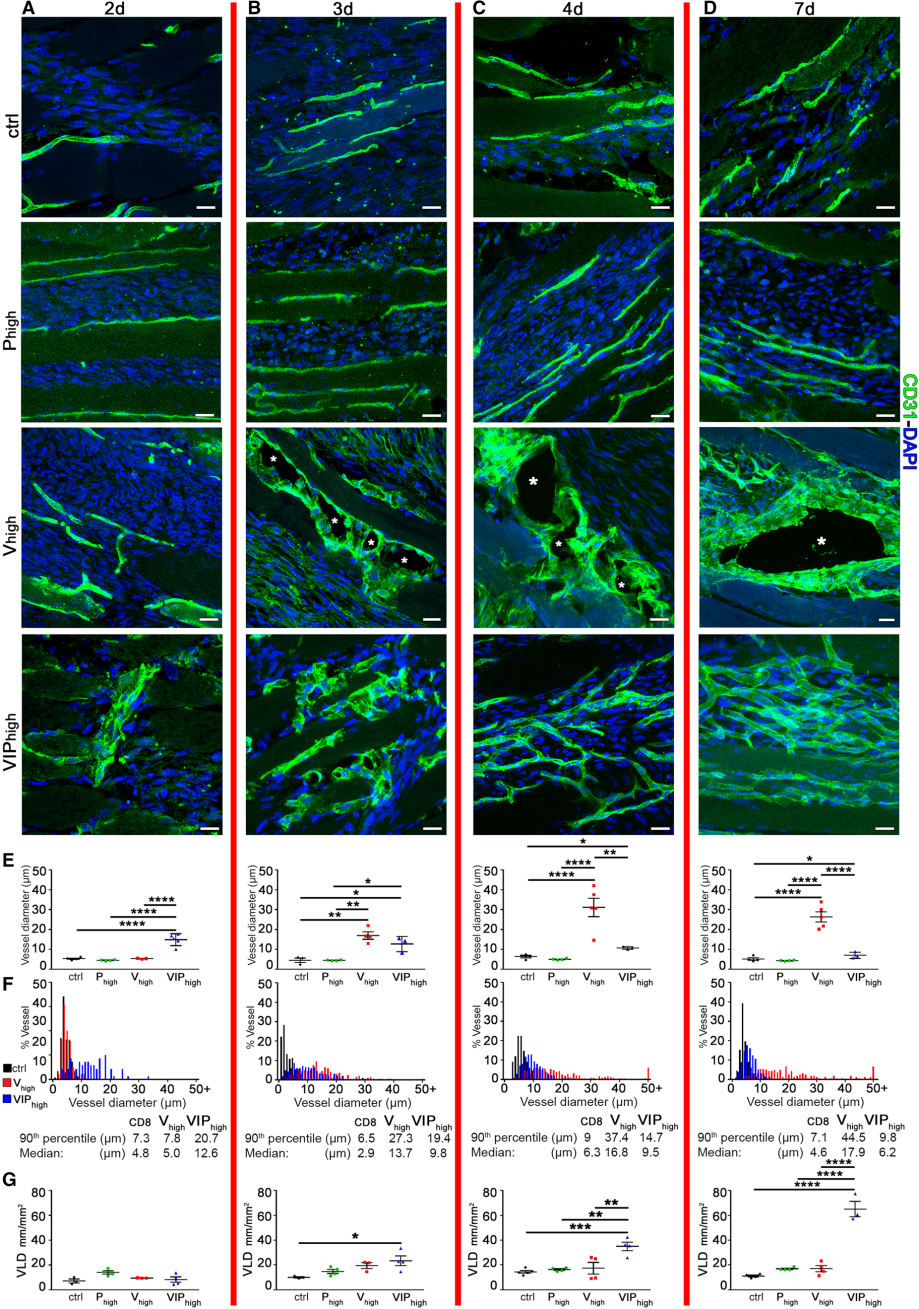
PDGF-BB co-expression limits the degree of vascular enlargement and induces robust normal angiogenesis. The time point labels (2, 3, 4, and 7 days) refer to all images and graphs in each column delimited by the red vertical lines. **a–d** Cryosections of TA and GC muscles implanted with *V*_high_, VIP_high_, *P*_high_, and control myoblasts were immunostained for CD31 (endothelial cells, green) and DAPI (nuclei, blue). **b**–**c** *Lumen of vascular enlargement and **d** of aberrant structure. **e** Values represent means (in µm) of individual measurements in each sample ± SEM quantified in areas of myoblast implantation at day 2, 3, 4, and 7 post-implantation. *p < 0.05, **p < 0.01, ****p < 0.0001 by 1-way ANOVA. **f** Distribution of vessel diameters (in µm). **g** The amount of angiogenesis was quantified in the same areas: *VLD* vessel length density, expressed as millimeters of vessel length per square millimeter of area of effect (mm/mm^2^). Data represent mean values ± SEM. *p < 0.05, **p < 0.01, ***p < 0.001, ****p < 0.0001 by 1-way ANOVA. *n* = 3–5 independent muscles per each group; per time point. Scale bars = 20 µm in all panels

We previously found that the switch between normal and aberrant angiogenesis correlates with a VEGF dose-dependent increase in the size of initial vascular enlargements [9]. Therefore we investigated whether PDGF-BB affected the circumferential enlargement induced by VEGF. Normal capillaries in areas implanted with control cells had a very homogeneous size that was stable over time (Fig. 3a–d, e, f). Clonal myoblasts producing only PDGF-BB at the same level as the VIP_high_ cells (*P*_high_) were implanted as a further control, showing that PDGF-BB alone did not affect pre-existing vessels over 7 days (Fig. 3a–d), causing no changes in either vessel size or quantity (Fig. 3e, g). High VEGF caused an enlargement of the pre-existing capillaries starting after 3 days and some of these vessels continued to grow in girth over time, failed to split and gave rise by 7 days to typical aberrant bulbous structures resembling angiomas, as evidenced both by the further increase in average diameter by 4 and 7 days (Fig. 3e; day 3 = 17.0 ± 1.8 µm; day 4 = 31.2 ± 4.7 µm and day 7 = 26.4 ± 2.6 µm) and by the appearance and persistence of a population of severely enlarged vessels > 30 µm in the diameter distribution analysis (Fig. 3f; day 4 = 18.8% and day 7 = 19.6%). On the other hand, PDGF-BB co-delivery caused vascular enlargements to start already by 2 days, with a size similar to that of high VEGF alone at 3 days (day 2 VIP_high_ mean diameter = 14.8 ± 1.3 µm). However, these vessels did not undergo any further increase in girth by 3 days and by 4 days they were actually mostly already remodeled into networks of normal capillaries (VIP_high_ mean diameter day 3 = 12.7 ± 2.2 µm, day 4 = 10.7 ± 0.3 µm and day 7 = 7.0 ± 0.9 µm; Fig. 3e) of homogeneous size, with a diameter distribution similar to that of control samples by day 7, with no vessels > 30 µm (Fig. 3f).

The amount of induced angiogenesis was quantified by measuring vessel length density (VLD), defined as the total length of vessels in a given area independently of their diameter. High VEGF alone did not cause a significant increase in VLD compared to controls or PDGF-BB alone at any time point (Fig. 3g), as growth took place mostly increasing the size of vascular structures rather than contributing new vessels. However, PDGF-BB co-delivery caused VLD to be already doubled compared to controls by 3 days (Fig. 3g; Ctrl = 10.0 ± 0.5 and VIP_high_ = 23.3 ± 3.9 mm/mm^2^; *p* < 0.05), reaching a sixfold increase by 7 days (Fig. 3g; Ctrl. = 11.1 ± 0.8 and VIP_high_ = 65.1 ± 6.2 mm/mm^2^; *p* < 0.0001).

Taken together, these results indicate that PDGF-BB co-expression (a) limits the degree of vascular enlargement induced by high levels of VEGF; and (b) enables completion of vascular splitting leading to robust generation of normal capillary networks.

### PDGF-BB limits sustained endothelial proliferation by high VEGF and does not cause fibrosis

**Fig. 4:**
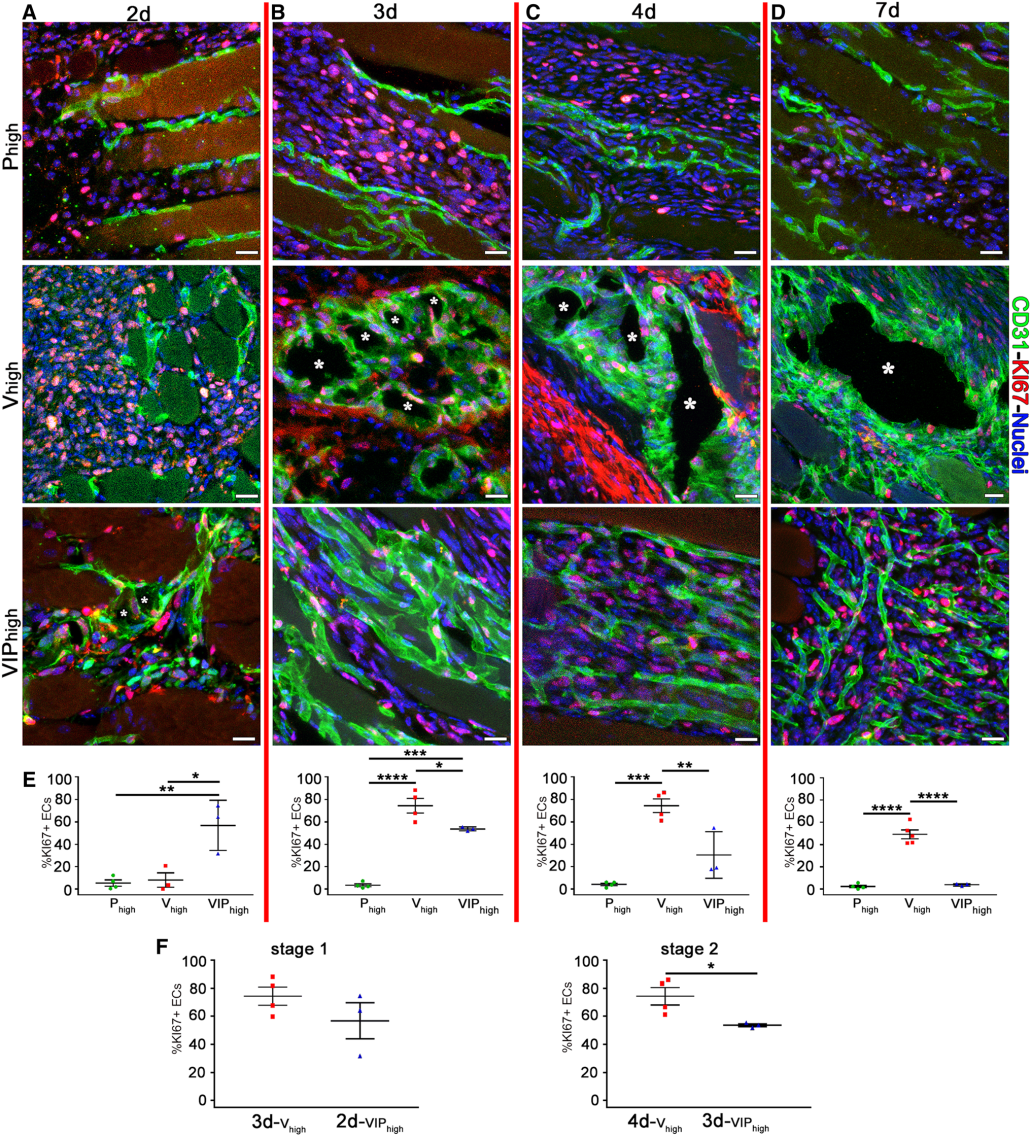
PDGF-BB co-expression limits endothelial proliferation. **a**–**d** Immunostaining with antibodies against CD31 (endothelial cells, green), Ki-67 (nuclei of proliferating cells, red), and with DAPI (nuclei, blue) was performed on cryosections of limb muscles injected with *V*_high_, VIP_high_, and *P*_high_ myoblast clones at day 2, 3, 4, and 7 after myoblast implantation. The time point labels (2, 3, 4, and 7 days) refer to all images and graphs in each column delimited by the red vertical lines. **a**–**c** *Lumen of vascular enlargements and **d** of aberrant angioma-like structure. **e**–**f** Quantification of KI67 marker in areas of effect showed that PDGFB co-expression reduced the total amount of proliferating endothelial cells compared to VEGF alone. Values represent means of individual measurements in each sample ± SEM. *p < 0.05, **p < 0.01, ***p < 0.001, ****p < 0.0001 by 1-way ANOVA. *n* = 3–5 independent muscles per each group, per time point. Scale bars = 20 µm in all panels

Next we asked whether PDGF-BB co-delivery could limit vascular enlargement by regulating endothelial proliferation induced by high VEGF. Immunostaining for Ki67 marks all proliferating cells in any phase of the cell cycle (G1-S-G2-M), but not quiescent ones in G0 [15]. Quantification of Ki67+ endothelial cells showed that PDGF-BB alone did not affect endothelial proliferation at any time point (Fig. 4a–e). High VEGF alone caused markedly increased endothelial proliferation by day 3 (74.4 ± 6.5%) and this was sustained through day 7 (day 4 = 74.4 ± 6.1% and day 7 = 49.2 ± 3.9%; Fig. 4a–e), consistently with the kinetics of vascular enlargement described above (Fig. 3e). On the other hand, PDGF-BB co-delivery caused endothelial proliferation to start already by day 2 (56.8 ± 12.9%), with no further increase by day 3 (53.7 ± 1.0%) and gradually subsiding by day 4 (30.8 ± 12.0%) to reach almost complete quiescence by day 7 (3.9 ± 0.6%; Fig. 4a–e). A comparison of proliferation at the 2 biologically equivalent stages defined above (Stage 1 = first day of enlargement, and Stage 2 = second day of enlargement) showed that PDGF-BB co-delivery caused a moderate decrease in the number of proliferating endothelial cells by about 15% during initial vascular enlargement (Fig. 4f). These results suggest that PDGF-BB co-expression can limit the degree of vascular enlargement and prevent aberrant angiogenesis both by moderately reducing the initial endothelial proliferation induced by high VEGF and rapidly abolishing it after day 4.

**Fig. 5:**
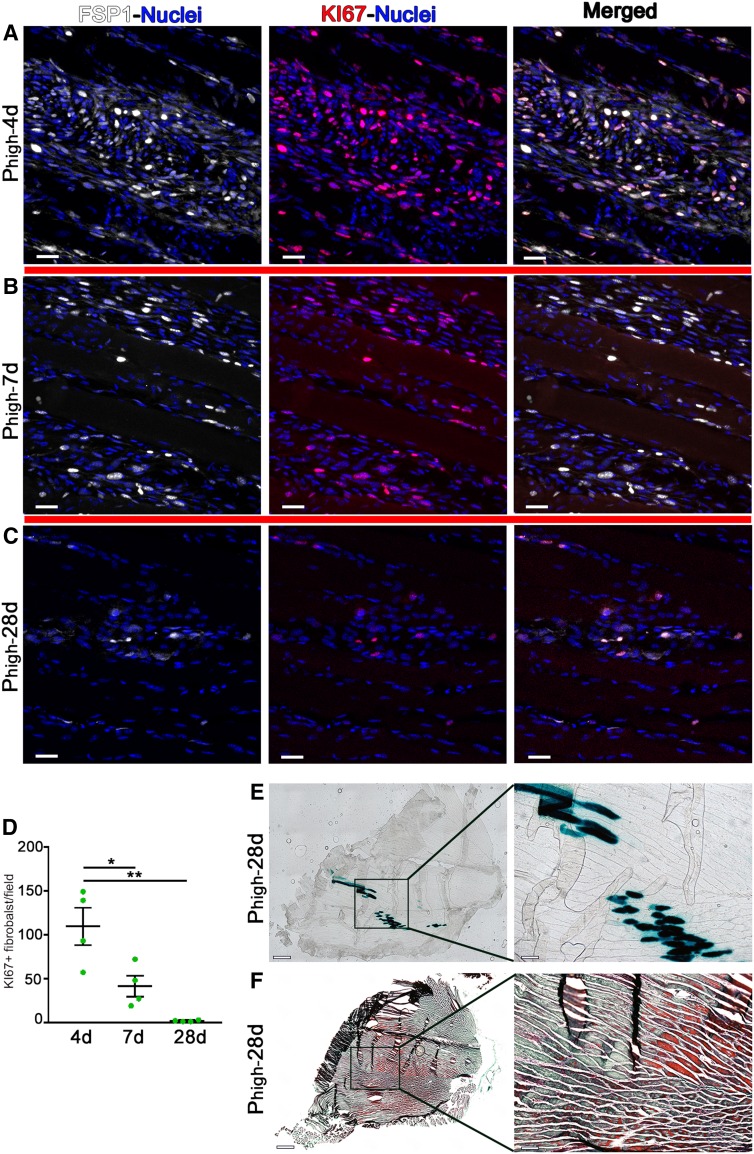
PDGF-BB over-expression does not cause fibrosis. **a**–**c** Immunostaining with antibodies against FSP1 (fibroblasts, white), Ki-67 (nuclei of proliferating cells, red), and with DAPI (nuclei, blue) was performed on cryosections of limb muscles injected with *P*_high_ myoblasts at day 4, 7, and 28 days after myoblast implantation. Scale bars = 20 µm. **d** Quantification of the number of Ki-67 + fibroblast/field of view in areas of effect showed no sustained fibroblast proliferation by 4 weeks. Values represent means ± SEM. *p < 0.05, **p < 0.01 by 1-way ANOVA. **e**–**f** X-Gal staining (**e**) and Masson trichrome staining (**f**) showed persistent engraftment of LacZ-expressing myoblasts and the absence of fibrosis, respectively, after 28 days. The panels on the right in **e** and **f** represent high-magnification views of the areas marked by black boxes in the left panels. Scale bars = 500 µm in low-magnification panels (left) and 100 µm in high-magnification panels (right). *n* = 4 muscles per time point

Since PDGF-BB is also a fibroblast mitogen [16], we investigated the possible induction of fibrosis. Co-staining for Ki-67 and the fibroblast-specific marker FSP1 [17] detected actively proliferating fibroblasts between muscle fibers in the areas of implantation of *P*_high_ myoblasts (Fig. 5a–c). However, fibroblast proliferation was transient between 4 and 7 days after myoblast implantation and by 28 days had subsided (4 days = 109.5 ± 21.3 Ki-67 + fibroblasts/field, 7 days = 41.5 ± 11.9 and 28 days = 1.7 ± 0.5; Fig. 5d), despite stable engraftment of the PDGF-BB-expressing myoblasts (Fig. 5e). In agreement with the lack of sustained fibroblast proliferation, no signs of fibrosis were detected in implanted muscles after 28 days (Fig. 5f).

### PDGF-BB prevents early pericyte loss induced by high VEGF

**Fig. 6:**
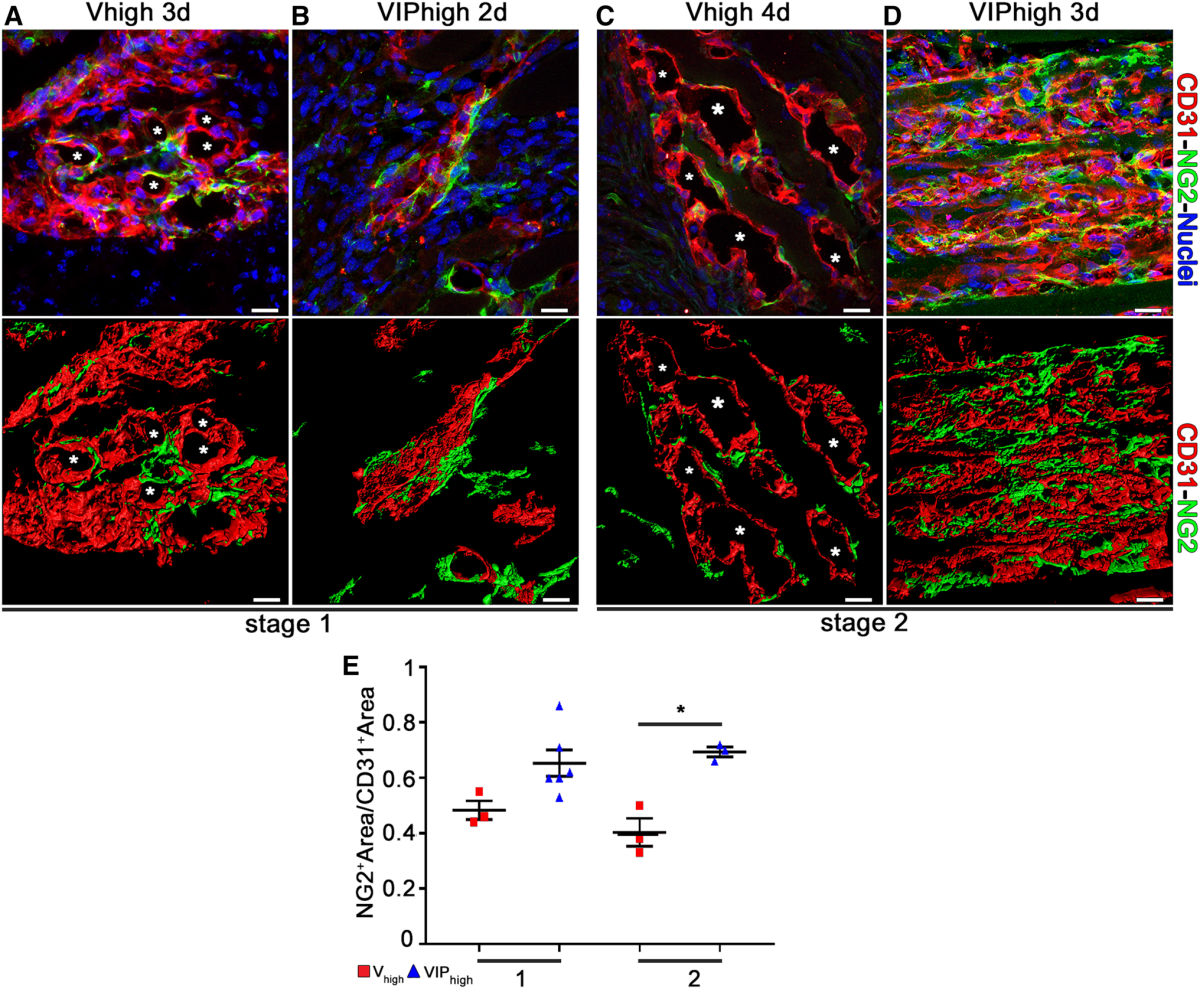
Mural cell coverage. **a**–**d** Vessels induced by implantation of *V*_High_ and VIP_High_ myoblast clones were immunostained with antibodies against CD31 (endothelial cells, red), NG2 (pericytes, green), α-SMA (smooth muscle cells, cyan), and with DAPI (nuclei, blue) in cryosections of TA and GC muscles. **a** and **c** *Lumen of vascular enlargements. **e** The maturation index was quantified in areas implanted with each cell population at stage 1 and 2 of VEGF-induced vascular enlargements. Co-expression of PDGF-BB caused a marked increase in mural cell coverage at both time points compared to high VEGF alone. Data represent mean values ± SEM; *p < 0.05 by Kruskal–Wallis test. *n* = 3–6 independent muscles per each group; per time point. Scale bars = 20 µm in all panels

We have previously found that high VEGF causes loss of vascular pericytes [9, 10]. Therefore, we sought to determine whether PDGF-BB co-expression could modulate the effects of high VEGF on pericyte loss during the initial stages of vascular enlargement. Pericyte coverage of vascular structures was quantified after implantation of *V*_high_ and VIP_high_ myoblast clones at Stage 1 and 2 of enlargement (days 3/2 and 4/3, respectively, for *V* and VIP) by measuring their maturation index, i.e., the ratio of the NG2+/CD31+ signal after immunostaining (Fig. 6  a–e). The results show that PDGF-BB co-expression prevented the loss of NG2 + pericytes both at stage 1 and 2, maintaining a more than 50% greater maturation index by Stage 2 (*V*_high_ = 0.4 ± 0.1 vs. VIP_high_ = 0.7 ± 0.0; *p < 0.05; Fig. 5e). Therefore, the normalization of aberrant vascular remodeling by PDGF-BB co-expression correlates with pericyte retention on the endothelial structures during the initial stages of VEGF-induced enlargement.

### PDGF-BB does not affect microhemodynamic changes during VEGF-induced non-sprouting angiogenesis

**Fig. 7:**
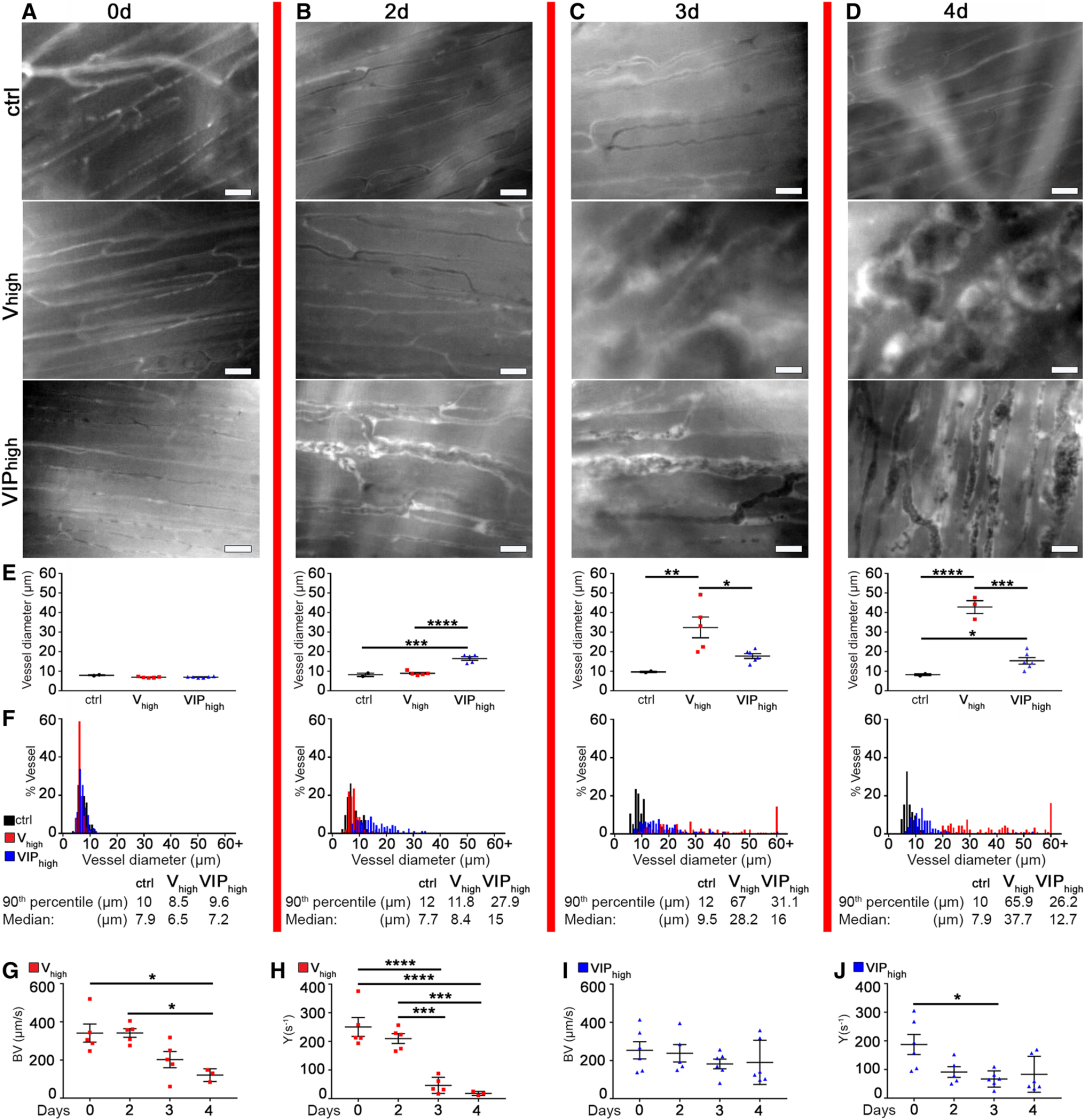
Microhemodynamics and vascular splitting. *V*_High_, VIP_High_, and control myoblasts were implanted into the panniculus carnosus muscle of SCID mice in a dorsal skinfold chamber. The time point labels (0, 2, 3, and 4 days) refer to all images and graphs in each column delimited by the red vertical lines. **a**–**d** Intravital fluorescence microscopic images of capillaries in areas of myoblast implantation. **e** Values represent means (in µm) of individual measurements in each sample ± SEM quantified in areas of myoblast implantation at day 0, 2, 3, and 4 post cell implantation. *p < 0.05, **p < 0.01, ***p < 0.001 ****p < 0.0001 by 1-way ANOVA. **f** The distribution of vessel diameters (in µm) was quantified in areas of myoblast implantation. **g**–**j** Blood flow velocity (BV, in µm/s) and wall shear rate (γ, in s^−1^) were quantified off-line concomitantly with onset of vascular enlargements. Data represent mean values ± SEM; *p < 0.05, *p < 0.05, ***p < 0.001, ****p < 0.0001 by 1-way ANOVA or by Kruskal–Wallis test. *n* = 2–6 independent muscles per each group; per time point. Scale bars = 100 µm in all panels

Acute increases in both blood flow and shear stress have been shown to be potent triggers of splitting angiogenesis in microvascular networks and to rapidly initiate transluminal pillar formation without the need for growth factor delivery [18, 19]. Therefore, we investigated hemodynamic parameters by repetitive intravital imaging of vascular structures over time. *V*_high_ and VIP_high_ clones, as well as control myoblasts, were over-infected with a retroviral vector to stably produce the red fluorescent protein DsRed, to allow their direct visualization during *in vivo* imaging and were implanted into the panniculus carnosus muscle of SCID mice in a dorsal skinfold chamber. Intravital fluorescence microscopy was performed once per day on day 0 immediately after cell implantation and from day 2 (when vascular enlargements first appeared) up to day 4 (when remodeling into normal capillaries by PDGF-BB co-expression was almost complete). As expected, in muscles injected with control cells, only normal-sized capillaries were found that were unchanged over time (Fig. 7a–d). With high VEGF, pre-existing normal capillaries were still not affected at day 2 and displayed a diameter distribution similar to those in sites implanted with control cells (ctrl mean diameter = 8.2 ± 0.8 µm vs. *V*_high_ = 8.9 ± 0.4 µm. *p* = n.s.; Fig. 7e, f), but significant enlargement could be observed at days 3 and 4 (Fig. 7a–f), in agreement with the histological data shown in Fig. 3. On the other hand, PDGF-BB co-expression caused enlargement of pre-existing capillaries to start already by day 2 (Fig. 7a–d), but limited their size to a lesser degree of enlargement compared to VEGF alone at days 3 and 4, also confirming the histological findings above (VIP_high_ day 2 mean diameter = 16.5 ± 0.9 µm vs. *V*_high_ day 3 = 32.4 ± 5.3 µm, *p* < 0.01; VIP_high_ day 3 = 17.8 ± 1.2 µm vs. *V*_high_ day 4 = 42.9 ± 3.3 µm, ***p < 0.0001; Fig. 7e, f). Quantification of hemodynamic stimuli revealed that high VEGF caused a marked reduction of both blood flow velocity and shear rate (BV and Y, respectively; Fig. 7g, h) at days 3 and 4 compared to day 0, concomitantly with the marked vascular enlargement. Upon PDGF-BB co-expression blood velocity remained stable or decreased slightly during the vascular enlargement phase compared to day 0 (Fig. 7i), whereas shear rate also significantly diminished over days 2 and 3 (Fig. 7j). These data suggest that a global reduction in shear stress accompanied the initiation of transluminal pillar formation during VEGF-induced splitting angiogenesis, regardless of PDGF-BB co-expression.

### PDGF-BB limits VEGF signaling activation

**Fig. 8:**
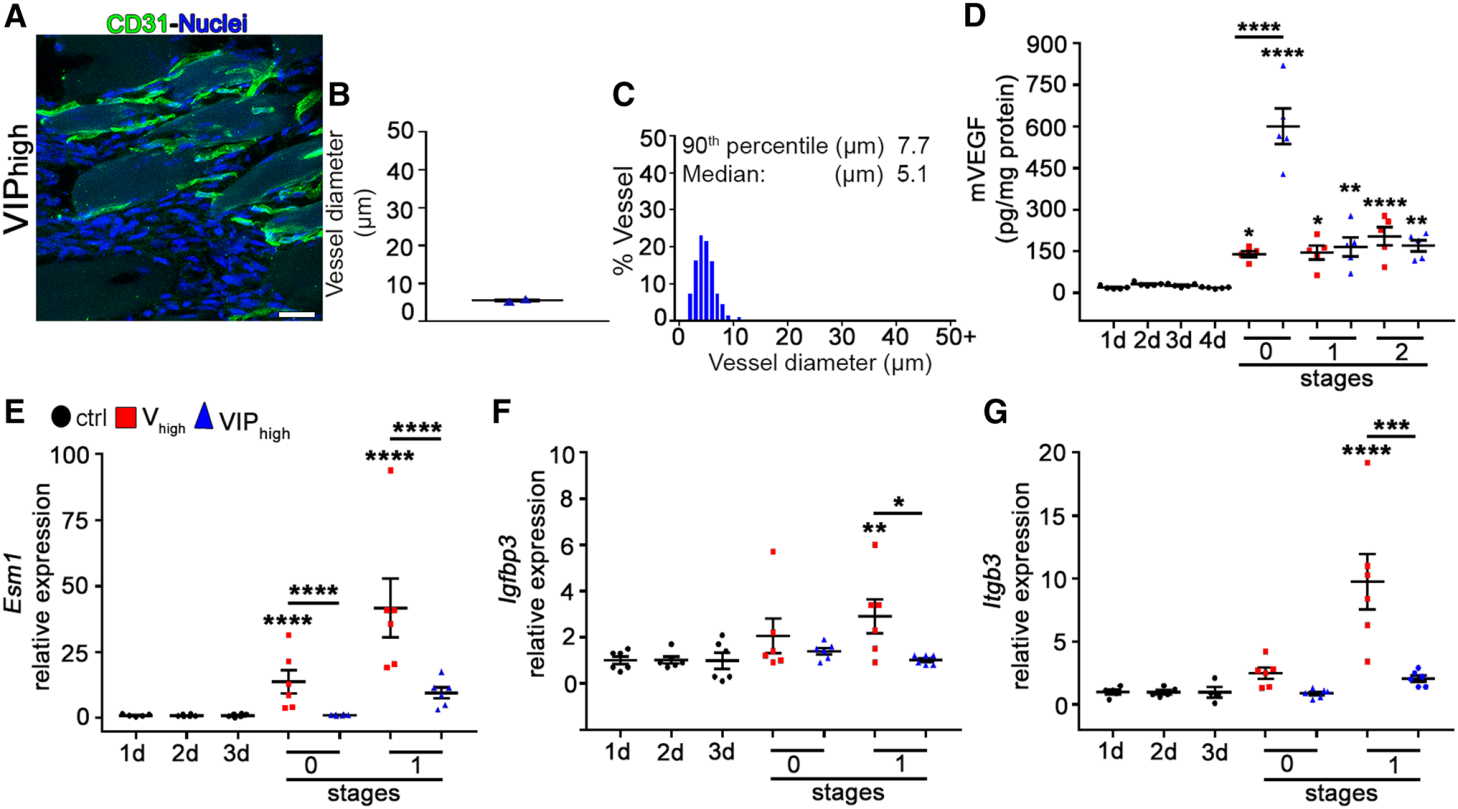
PDGF-BB co-expression reduces the activation of the VEGF signaling pathway. **a**–**c** Cryosections of TA and GC muscles implanted with VIP_high_ were immunostained for CD31 (endothelial cells, green) and DAPI (nuclei, blue). **a** 1 day after cell implantation the pre-existing vessels were not yet affected. **b** Values represent diameter mean (in µm) of individual measurements in each sample ± SEM quantified in areas of myoblast implantation. **c** Distribution of vessel diameters (in µm). *n* = 2 muscles. Scale bar = 20 µm. **d** Total VEGF protein content of muscle extracts was measured by ELISA (pg/mg of total protein). Data represent mean values ± SEM; *p < 0.05, **p < 0.01, ****p < 0.0001 by 1-way ANOVA. *n* = 5 independent muscles per group, per time point. **e**–**g** In vivo expression of *Esm1, Igfbp3*, and *Itgb3* genes, which are specifically induced by activation of VEGF-R2 signaling, was quantified in calf muscles at day 1, 2, and 3 after injections of control, V_high_, and VIP_high_ myoblasts. PDGF-BB co-expression significantly reduced the signaling output downstream of VEGF-R2, despite similar or even higher levels of VEGF protein in the tissues. *p < 0.05, **p < 0.01, ***p < 0.001, ****p < 0.0001 by 1-way ANOVA. *n* = 4–6 independent muscles per group, per time point

The biological effects of VEGF are transduced by the receptor tyrosine kinase VEGF-Receptor 2 (VEGF-R2) [20]. In order to determine whether PDGF-BB could modulate VEGF activity, we assessed the relative activation of VEGF-R2 signaling output by measuring the expression of its specific target genes endocan (endothelial-specific molecule-1, *Esm1*) [21], *Igfbp3* [22], and integrin β3 (*Itgb3*) [23]. The VIP_high_ clone, *V*_high_ clone, and control cells were injected in the tibialis anterior and gastrocnemius muscles of SCID mice. Gene expression was measured at stage 1 of vessel development, as defined above, when enlarged vessels started to appear in treated muscles (day 3 for *V*_high_ and day 2 for VIP_high_), as well as on the preceding day, defined as stage 0 immediately before the start of morphogenic events (day 2 for *V*_high_ and day 1 for VIP_high_). This time point was added because at this stage vascular enlargements are not yet induced, but differences in VEGF-R2 signaling might be already established that lead to subsequent changes. In order to be sure that VIP-induced changes did not start earlier than day 2, muscles were analyzed 1 day after implantation with VIP_high_ cells (Fig. 8a–c). Capillaries in the areas of implantation did not show any signs of circumferential enlargement, as vessel diameters were similar to those of the day 2 control samples analyzed in Fig. 3e, f (mean = 5.2 ± 0.2 µm, median = 5.1 µm and 90th percentile = 7.7 µm). Further, we verified the possibility that in vivo VEGF expression by the VIP_high_ clone could be lost faster than by the *V*_high_ clone after implantation, which could be a confounding factor. ELISA quantification of VEGF protein in total muscles harvested from stages 0, 1, and 2 showed that, as expected, VEGF production was increased in all groups compared to control tissues. However, no loss of VEGF production could be seen in VIP-implanted muscles, which actually contained an even higher amount of VEGF than *V*_high_ samples at stage 0 and a similar one afterwards (Fig. 8d).

As shown in Fig. 8e–g, VEGF alone up-regulated the expression of all 3 target genes at stage 1 and *Esm1* already at stage 0. On the other hand, in the presence of PDGF-BB, the signaling output of VEGF-R2 appeared significantly reduced compared to VEGF alone in all instances, as evidenced by significantly lower expression of all target genes at stage 1, as well as of *Esm-1* also at stage 0.

Taken together, these findings suggest that PDGF-BB co-expression limited the degree of enlargement of pre-existing capillaries by reducing the activation of VEGF-R2 signaling in the endothelium compared to VEGF alone.

## Discussion

Taking advantage of a highly controlled myoblast-based gene delivery platform, here we found that PDGF-BB normalizes aberrant angiogenesis by high levels of VEGF in the therapeutic target tissue of skeletal muscle by enabling efficient vascular splitting and without inducing any sprouting. Hemodynamic parameters, such as blood velocity and shear stress, were not responsible for the effective completion of splitting. Rather, PDGF-BB co-delivery prevented the excessive enlargement of the pre-existing vessels, thereby enabling efficient and accelerated completion of vascular splitting into normal capillaries already by 4 days after factor delivery. Mechanistically, PDGF-BB moderated the degree of endothelial proliferation by limiting the VEGF-R2 signaling output by similar VEGF doses.

It is important to recognize that angiogenesis in skeletal muscle can occur through two alternative mechanisms: sprouting, which is mainly initiated by hypoxia during ischemia [24] and splitting [14, 25]. Sprouting relies on the specification of specialized endothelial tip cells, which migrate into surrounding tissue and are followed by proliferating stalk cells [13]. On the other hand, splitting angiogenesis takes place without any abluminal endothelial migration and comprises two different cellular mechanisms: (1) intussusception, which relies on the formation of transluminal pillars through a zone of endothelial contact between invaginations of the opposite vessel walls [14]; and (2) longitudinal division, where exclusively endothelial filopodial ridges are extended intraluminally, without involvement of the vessel wall [25]. Although it is not yet clear whether these two processes take place in different kinds of vessels, both lead to the generation of new vascular segments by vascular splitting rather than by abluminal sprouting; for the sake of clarity, here we use the term “vascular splitting” to comprise both. Recently, we found that VEGF delivery to skeletal muscle at therapeutically relevant doses induces both normal and aberrant angiogenesis essentially without sprouting, but rather through a first stage of circumferential enlargement followed by longitudinal splitting [9]. Here, we now show that PDGF-BB co-delivery prevents aberrant vascular growth by high VEGF doses, converting it into normal microvascular networks, by modulating the same mechanism, i.e., by enabling efficient and accelerated vascular splitting, and not by switching to a sprouting mode of angiogenesis.

It has been previously shown that splitting angiogenesis in skeletal muscle can be initiated by changes in haemodynamic factors [26]. In fact, sustained increases in blood flow and capillary shear stress, caused by chronic vasodilation with the α-adrenergic inhibitor prazosin, induced capillary growth by longitudinal division in rodent muscle [19, 26–28]. Further, in the chicken chorioallantoic membrane (CAM) acute increases in flow and shear stress, achieved by clamping one of the major arterial side-branches upstream of the investigated areas, also caused many of the vascular bifurcations within these areas to undergo intussusceptive remodeling within 15–45 min [18]. On the other hand, here we found that high VEGF initially caused a marked global reduction of both blood flow velocity and shear stress, likely due to the marked enlargement of vessel diameters. However, PDGF-BB co-delivery did not change the trend of both hemodynamic parameters, which behaved similarly to the condition with VEGF alone. In agreement with these findings, the frequency of pillar formation was not increased by PDGF-BB co-delivery compared to VEGF alone (Fig. 1i), suggesting that the increased efficiency of vascular splitting by PDGF-BB was not mediated through hemodynamic regulation.

The apparent discrepancy with the results of vasodilation and clamping experiments can likely be explained by considering that in those conditions no growth factor was delivered and hemodynamic parameters were modified in the absence of any vascular enlargement. In contrast, the first effect of VEGF delivery to skeletal muscle is a circumferential enlargement of pre-existing vessels mediated by endothelial proliferation, which rather leads to a global decrease in flow and shear stress, as expected. Interestingly, recent data generated by 3D computational flow simulations based on in vivo calculations of haemodynamics parameters showed that, within single vessels, transluminal pillars were located in regions of comparatively lower shear stress and were spatially constrained by neighboring regions of higher flow and shear stress [29, 30]. Therefore, further insights into the fine regulation of pillar initiation by hemodynamic changes under therapeutically relevant conditions of growth factor delivery will require ad hoc investigations of intra-vascular distribution of flow and shear in single vessels.

On the other hand, the key effect of PDGF-BB co-delivery was a limitation of the size of vascular enlargements induced by VEGF through a reduction in endothelial proliferation. This observation is consistent with previous findings in developmental angiogenesis. In fact, the severe impairment in pericyte recruitment caused by genetic ablation of the *Pdgfb* gene was shown to cause unabated endothelial proliferation, leading to the formation of aberrant microaneurysms and lethal hemorrhages [31]. Conversely, PDGF-BB over-expression and increased pericyte recruitment were found to limit excessive tumor angiogenesis by inhibiting endothelial proliferation [32]. In the context of VEGF over-expression, the observed limitation of endothelial proliferation provides a plausible mechanism for the successful splitting into normal capillaries enabled by PDGF-BB co-delivery, despite stimulation with high VEGF levels. In fact, we have previously found that the size of the initial vascular enlargements depends on the VEGF dose and, while in the smaller vessels induced by low VEGF pillar formation could proceed successfully and complete the splitting, in the larger-caliber structures induced by high VEGF pillar formation would start, but it could not be completed due to the excessive vascular diameter to bridge, leading to a failure to split and the continued circumferential growth of affected segments into angioma-like structures [9]. Here, we show that PDGF-BB co-delivery limits the size of initial enlargements caused by high VEGF, leading to diameters more similar to those induced by low VEGF alone [9]. These smaller-caliber vascular enlargements could successfully complete vascular splitting, even though the density of initial pillar formation was not increased compared to VEGF alone.

As previously described [9], enlarged vessels induced by high VEGF displayed a marked loss of pericyte coverage. In fact, VEGF at high doses can interfere with PDGF-BB signaling and negatively regulate pericyte recruitment [33] and we have previously found that endogenous PDGF-BB expression does not increase with increasing doses of VEGF delivery, leading to an imbalance between the two signaling pathways, loss of pericytes, and the switch to aberrant angiogenesis [10]. Accordingly, PDGF-BB co-delivery could prevent both pericyte loss and aberrant angiogenesis [10], in agreement with the findings reported here.

Pericytes establish complex regulatory functions on endothelial cells through both paracrine and juxtacrine signaling, among which the main pathways include Transforming-Growth Factor-β, angiopoietin-1/-2, and ephrinB2/EphB4 [34]. For example, TGF-β1 can inhibit endothelial proliferation and promote vessel stabilization [35], whereas ephrinB2 has been shown to regulate VEGF-R2 internalization and its loss reduces VEGF-R2 signaling [36]. Interestingly, we recently found that EphB4 stimulation by ephrinB2 fine-tunes the degree of endothelial proliferation induced by specific VEGF doses through ERK1/2 modulation, thereby limiting the size of vessel enlargement and enabling successful splitting [37]. As ephrinB2 is expressed by pericytes and requires cell-to-cell contact to engage and activate EphB4 [38], PDGF-BB might regulate endothelial proliferation by promoting pericyte-endothelial crosstalk through ephrinB2/EphB4 signaling, although a cell-autonomous action on endothelium cannot be excluded. Our data indicate that the limitation of VEGF-R2 signaling output by PDGF-BB co-expression has significant therapeutic implications, by efficiently preventing aberrant angiogenesis despite high VEGF doses, without interfering with normal microvascular growth.

## Methods

### Cell culture

Primary myoblasts isolated from C57BL/6 mice and transduced to express the β-galactosidase marker gene (lacZ) from a retroviral promoter [39] were over-infected at high efficiency [40] with the retroviruses expressing murine VEGF_164_ alone (V) or together with human PDGF-BB at a fixed ratio to each other from a bicistronic cassette through the encephalomyocarditis virus Internal Ribosomal Entry Site (IRES) as previously described (VIP: Vegf-Ires-Pdgfb) [10]. A truncated version of CD8a (trCD8a) was co-expressed with VEGF from a similar bicistronic cassette (V), or from a separate promoter (VIP), as a convenient cell surface marker of transduced cells, as described [11]. Single cells were isolated from the polyclonal myoblast populations V, and VIP by FACS, based on their CD8 staining, using a Vantage SE cell sorter (Becton Dickinson, Basel, Switzerland). Single-cell isolation was confirmed visually and monoclonal populations of transduced myoblasts were expanded in culture to express specific and homogeneous levels of each factor, as previously described [6, 7, 10, 11]. All myoblast populations were cultured in 5% CO_2_ on collagen-coated dishes, with a growth medium consisting of 40% F10, 40% DMEM low glucose (1000 mg glucose/l) (Sigma-Aldrich Chemie GmbH, Steinheim, Germany), and 20% fetal bovine serum (HyClone, Logan, UT) supplemented with 2.5 ng/ml basic fibroblast growth factor (FGF-2) (Becton Dickinson, Basel, Switzerland), as described [41].

### Myoblast implantation into mice

Cells were implanted into 8–24-week-old immunodeficient SCID CB.17 mice (Charles River Laboratories, Sulzfeld, Germany) in order to avoid an immunological response to β-galactosidase-expressing myoblasts and human *Pdgfb*. Animals were treated (a) in accordance with Swiss Federal guidelines for animal welfare and the study protocol was approved by the Veterinary Office of the Canton Basel-Stadt (Basel, Switzerland) and (b) in accordance with the guidelines for the Care and Use of Laboratory Animals and the Institutional Animal Care and Use Committee (University of Rostock, Medical Faculty, Rostock, Germany). Myoblasts were dissociated in trypsin and resuspended at a concentration of 10^8^ cells/ml in sterile PBS with 0.5% BSA. 1 × 10^6^ cells in 10 µl were implanted into the tibialis anterior (TA) and gastrocnemius (GC) muscles of the leg as previously described [7], or into the Panniculus Carnosus muscle, i.e., the thin subcutaneous muscle layer of murine skin, by using a syringe with a 29^1^/_2_G needle.

### Vascular casting

Vascular casts were prepared as previously described [42]. Briefly, the vasculature was perfused with a freshly prepared solution of PU4ii polymer (vasQtec, Zurich, Switzerland). One hour after perfusion, the samples were transferred to 7.5% potassium hydroxide for dissolution of tissue, which was completed over 2–3 weeks. After washing, the casts were freeze-dried and glued onto the aluminum sample stabs. The samples were then sputtered with gold to a thickness of 10 nm and examined in a Philips XL-30 SFEG scanning electron microscope. TA muscle received 1 cell injection, whereas GC muscle received 2 cell injections (in both the medialis and lateralis portions). At least 7 areas of clear angiogenic effect in 3 entire legs per group per time point were analyzed.

### Immunofluorescence tissue staining

Mice were anesthetized and the tissues were fixed by vascular perfusion of 1% paraformaldehyde in PBS pH 7.4 for 4 min under 120 mm/Hg of pressure, followed by 2 h of post-fixation in 0.5% paraformaldehyde in PBS pH 7.4. Entire tibialis anterior and gastrocnemius muscles excised from perfused mice were fixed for additional 2 h in 0.5% paraformaldehyde and then immersed overnight in 30% sucrose solution for cryoprotection before being embedded in OCT compound and frozen in freezing isopentane (Medite, Basel, Switzerland). Tissue sections were then stained with X-gal (20 µm sections) to reveal myoblast engraftment or with H&E (14 µm sections) as described previously [39, 40]. In addition, the presence of fibrosis was examined with Masson trichrome staining (Réactifs RAL, Martillac, France) according to manufacturer’s instructions. Immunofluorescence staining was performed on 20-µm-thick frozen sections of muscles tissues, cut along the longitudinal axis. The following primary antibodies and dilutions were used: rat anti-CD31 (clone MEC 13.3, BD Biosciences, Basel, Switzerland) at 1:100; rat anti-endomucin (clone V.7C7, Santa Cruz Biotechnology, Santa Cruz, CA); mouse anti-Mts1 (FSP-1) (clone X9-7, Santa Cruz Biotechnology, Santa Cruz, CA) at 1:200; mouse anti-α-SMA (clone 1A4, MP Biomedicals, Basel, Switzerland) at 1:400; rabbit anti-NG2 (Chemicon International, Hampshire, UK) at 1:200; chicken anti-laminin (Abcam, Cambridge, UK); rabbit anti-Ki67 (Abcam, Cambridge, UK) at 1:100; rat anti-Ki67 (clone SolA15, Invitrogen, Basel, Switzerland) at 1:200. Fluorescently labeled secondary antibodies (Invitrogen, Basel, Switzerland) were used at 1:200.

### Vessel analyses

Vessel length density (VLD) and diameters were quantified in fluorescently immunostained cryosections as previously described [7]. Briefly, VLD was measured in at least 3 fields/section per muscle, cut at 100–150 µm of distance from each other (*n* = 2–5 muscles/group) by tracing the total length of vessels in the field and dividing it by the area of the fields.

Vessel diameters were measured in fluorescently immunostained sections as described [7]. Briefly, 10–20 fields/muscle (*n* = 2–5 muscles/group) were analyzed, measuring a total of 100 up to 500 vessel diameter per time point by overlaying captured microscopic images with a square grid. Squares were randomly chosen, and the diameter of each vessel (if any) in the center of selected squares was measured. To avoid selection bias, the shortest diameter in the selected vascular segment was systematically measured. Ki67+ endothelial cells were quantified as a percentage of all endothelial cells in analyzed vascular structures: 400–3500 total endothelial cells were counted per condition and per time point taking 5–15 random fields of view/sample with a clear angiogenic effect (*n* = 3–5 muscles/group). Ki67+ fibroblasts were quantified in areas of myoblast implantation, detected by LacZ staining: 40–1900 total fibroblasts were counted per time point and condition taking 5–10 random fields of view/sample (*n* = 4 muscles/group). Fluorescence images were acquired as Z-Stack with ×40 objective on a Carl Zeiss LSM710 3-laser scanning confocal microscope (Carl Zeiss, Feldbach, Switzerland). 3-D immunofluorescence images were generated by using Imaris 7.6.5 software (Bitplane, Zürich, Switzerland). All image measurements were performed with LSM software Zen 2010 (Carl Zeiss, Feldbach, Switzerland) or with cellSens software (Olympus, Volketswil, Switzerland). The quantification of pericyte coverage was performed on sections of leg muscles after immunostaining for endothelium (CD31) and pericytes (NG2). The CD31- and NG2-positive areas were separately subjected to threshold processing. The areas occupied by their respective signals were measured by Imaris 7.6.5 software (Bitplane, Zürich, Switzerland) on Z-Stack, 1024 × 1024, 8 bit Fluorescence images acquired with ×40 objective on a Carl Zeiss LSM710 3-laser scanning confocal microscope (Carl Zeiss, Feldbach, Switzerland), and the pericyte coverage index was calculated as the ratio between the two values.

### Dorsal skinfold chamber and vessel analyses

The dorsal skinfold chamber was prepared as originally described in mice [43]. Mice were anesthetized by an intraperitoneal injection of ketamine (90 mg/kg bw) and xylazine (25 mg/kg bw) and placed on a 37 °C heating pad. Briefly, a double skin layer on the back of the animal was implanted between two symmetric titanium frames. One skin layer was then completely removed in a circular area of 15 mm in diameter, and the remaining layers (consisting of striated skin muscle, subcutaneous tissue, and skin) were covered with a glass coverslip incorporated into one of the titanium frames. Animals showed no signs of discomfort or changes of sleeping and feeding habits. To reduce surgical trauma-associated deterioration of the chamber microcirculation, mice were allowed a recovery period of 2 days after implantation of the chamber. After injection of 0.1 ml fluorescein isothiocyanate (FITC)-labeled dextran (2%; molecular weight 150 kDa; Sigma-Aldrich, Munich, Germany) into the retro-orbital venous plexus and subsequent circulation for 30 s, microcirculation of the striated muscle tissue was visualized by intravital fluorescence microscopy using a Zeiss microscope (Axiotech vario; Zeiss, Jena, Germany). The microscopic procedure was performed at a constant room temperature of 21–23 °C. The epi-illumination setup included a 100-W HBO mercury lamp (Osram GmbH, Munich, Germany) and a blue filter system (450–490/ > 520 nm excitation/emission wavelength). Microscopic images were recorded by a charge-coupled device video camera (FK 6990 IQ-S; Piper, Schwerte, Germany) and stored on videotapes (Fujifilm video cassette; Fuji Magnet-ics GmbH, Düsseldorf, Germany). Microcirculatory variables were quantified off-line by analysis of the aforementioned videotaped images using a computer-assisted image analysis system (CapImage; Zeintl Software, Heidelberg, Germany). Functional Capillary Density, i.e., the length of all perfused capillaries per region of interest was normalized by the initial values measured per each experimental condition at day 0 immediately after myoblast implantation. Measurement of vascular wall shear rates (*Y*) was based on the Newtonian definition γ = 8 × v/ds^−1^, where d represents the individual inner vessel diameter and v represents the red blood cell centerline velocity divided by 1.6 according to the Baker–Wayland factor to correct the parabolic velocity profile in microvessels [44] (*n* = 2–5 muscles/group/time point).

### RNA extraction and quantitative real-time PCR

For RNA extraction from total muscles, freshly harvested tissue was frozen in liquid nitrogen and disrupted using a Qiagen TissueLyser (Qiagen, Basel, Switzerland) in 1 ml TRIzol reagent (Invitrogen, Basel, Switzerland) for 100 mg of tissue. Total RNA was isolated from lysed tissues with the mirVANA Kit (Life Technologies, Zug, Switzerland) according to manufacturer’s instruction. RNA was reverse transcribed into cDNA using the OmniScript Reverse Transcription kit (Qiagen, Basel, Switzerland) at 37 °C for 60 min. Quantitative Real-Time PCR (qRT-PCR) was performed on an ABI 7500 Real-Time PCR system (Applied Biosystems, Basel, Switzerland). Expression of genes of interest was determined using the following TaqMan Gene Expression assays (Applied Biosystems, Basel, Switzerland) according to manufacturer’s instructions: mouse *Gapdh* (Mm03302249_g1), mouse *Esm1* (Mm00469953_m1), mouse *Igfbp3* (Mm01187817_m1), and mouse *Itgb3* (Mm00443980_m1). The cycling parameters were 50 °C for 2 min, followed by 95 °C for 10 min, and 40 cycles of denaturation at 95 °C for 15 s and annealing/extension at 60 °C for 1 min. Reactions were performed in triplicate for each template, averaged, and normalized to expression of the Gapdh housekeeping gene at each specific corresponding time point.

### VEGF protein and total RNA assay in muscle tissue

Whole fresh mouse muscles were disrupted using a Qiagen TissueLyser (Qiagen, Hombrechtikon, Switzerland) in 1 ml of PBS + 1% Triton X-100 for 100 mg of tissue, supplemented with Complete Protease Inhibitor Cocktail (Roche Diagnostics, Rotkreuz, Switzerland), which was non-denaturing for proteins. After centrifugation, lysates were used for VEGF protein quantification by ELISA analysis (R&D Systems Europe, Abingdon, UK) normalized for the for total extracted protein/muscle quantified by using the Advanced Protein Assay (Sigma-Aldrich Chemie GmbH, Steinheim, Germany). (*n* = 5 muscles per group per time point).

### Statistical analysis

Data are presented as mean ± standard error. The significance of differences was assessed with the GraphPad Prism 7.03 software (GraphPad Software). The normal distribution of all data sets was tested by D’Agostino and Pearson or Shapiro–Wilk and, depending on the results, multiple comparisons were performed with the parametric 1-way analysis of variance (ANOVA) followed by the Tukey or Sidak test for multiple comparisons, or with the non-parametric Kruskal–Wallis test followed by Dunn’s post-test, while single comparisons were analyzed with the non-parametric Mann–Whitney test. Gene expression data representing fold-changes vs control, which are asymmetrically distributed, were first normalized by logarithmic transformation and then analyzed by 1-way ANOVA followed by the Sidak test for multiple comparisons. Vessel diameter values were first normalized by log2-transformation and then analyzed by 1-way ANOVA followed by the Tukey test for multiple comparisons. *p* < 0.05 was considered statistically significant.
